# Predictors Associated with Increase in Skeletal Muscle Mass after Sustained Virological Response in Chronic Hepatitis C Treated with Direct Acting Antivirals

**DOI:** 10.3390/nu9101135

**Published:** 2017-10-18

**Authors:** Kazunori Yoh, Hiroki Nishikawa, Hirayuki Enomoto, Akio Ishii, Yoshinori Iwata, Yuho Miyamoto, Noriko Ishii, Yukihisa Yuri, Kunihiro Hasegawa, Chikage Nakano, Takashi Nishimura, Nobuhiro Aizawa, Yoshiyuki Sakai, Naoto Ikeda, Tomoyuki Takashima, Ryo Takata, Hiroko Iijima, Shuhei Nishiguchi

**Affiliations:** Division of Hepatobiliary and Pancreatic Disease, Department of Internal Medicine, Hyogo College of Medicine, Hyogo 663-8501, Japan; mm2wintwin@ybb.ne.jp (K.Y.); hi-nishikawa@hyo-med.ac.jp (H.N.); akio0010@yahoo.co.jp (A.I.); yo-iwata@hyo-med.ac.jp (Y.I.); yuho.0818.1989@gmail.com (Y.M.); ishinori1985@yahoo.co.jp (N.I.); gyma27ijo04td@gmail.com (Y.Y.); hiro.red1230@gmail.com (K.H.); chikage@hyo-med.ac.jp (C.N.); tk-nishimura@hyo-med.ac.jp (T.N.); nobu23hiro@yahoo.co.jp (N.A.); sakai429@hyo-med.ac.jp (Y.S.); nikeneko@hyo-med.ac.jp (N.I.); tomo0204@yahoo.co.jp (T.T.); chano_chano_rt@yahoo.co.jp (R.T.); hiroko-i@hyo-med.ac.jp (H.I.); nishiguc@hyo-med.ac.jp (S.N.)

**Keywords:** chronic hepatitis C, direct acting antiviral, sustained virological response, skeletal muscle mass

## Abstract

Aims: We aimed to examine changes in skeletal muscle mass in chronic hepatitis C (CHC) patients undergoing interferon (IFN)-free direct acting antivirals (DAAs) therapy who achieved sustained virological response (SVR). Patients and methods: A total of 69 CHC patients treated with DAAs were analyzed. We compared the changes in skeletal muscle index (SMI) using bio-impedance analysis at baseline and SMI at SVR. SMI was calculated as the sum of skeletal muscle mass in upper and lower extremities divided by height squared (cm^2^/m^2^). Further, we identified pretreatment parameters contributing to the increased SMI at SVR. Results: SMI in males at baseline ranged from 6.73 to 9.08 cm^2^/m^2^ (median, 7.65 cm^2^/m^2^), while that in females ranged from 4.45 to 7.27 cm^2^/m^2^ (median, 5.81 cm^2^/m^2^). At SVR, 36 patients (52.2%) had increased SMI as compared with baseline. In the univariate analysis, age (*p* = 0.0392), hyaluronic acid (*p* = 0.0143), and branched-chain amino acid to tyrosine ratio (BTR) (*p* = 0.0024) were significant pretreatment factors linked to increased SMI at SVR. In the multivariate analysis, only BTR was an independent predictor linked to the increased SMI at SVR (*p* = 0.0488). Conclusion: Pretreatment BTR level can be helpful for predicting increased SMI after SVR in CHC patients undergoing IFN-free DAAs therapy.

## 1. Introduction

The ultimate goal of treatment for chronic hepatitis C (CHC) is to eliminate the hepatitis C virus (HCV) and thereby to suppress the liver disease progression and the liver carcinogenesis [1,2,3]. In cases with sustained virological response (SVR), the incidence of disease progression or carcinogenesis has been reported to be markedly decreased [1,2,4,5]. CHC therapy has dramatically changed with the recent accessibility of direct acting antivirals (DAAs). Protease inhibitors including telaprevir, simeprevir, or vaniprevir containing pegylated-interferon (Peg-IFN)α2a or Peg-IFNα2b and ribavirin (RBV) combination therapy (IFN-based triple therapy) have demonstrated higher SVR rates [6]. Further, IFN-free DAAs therapies such as daclatasvir (DCV)/asunaprevir (ASV), sofosbuvir (SOF)/RBV, ledipasvir (LDV)/SOF, LDV/SOF/RBV, and SOF/velpatasvir have also demonstrated excellent SVR rates [4,5,7,8]. Recently, almost all CHC patients have been able to eradicate HCV in a comfortable manner [3].

On the other hand, skeletal muscle loss (SML) has been shown to be an adverse predictor in patients with liver diseases and has drawn much caution among clinicians because of its linkage to clinical outcomes [9,10,11]. Skeletal muscle mass is maintained by a balance between protein synthesis and protein breakdown, and muscle mass loss can occur due to an increment in proteolysis or a reduction in protein synthesis, or both disorders [12,13,14]. There have been compelling evidences to show that SML is one of the major complications in liver cirrhosis (LC) due to the LC-related double metabolic burdens (i.e., protein metabolic and energy metabolic disorders) [11,12,15,16,17,18,19,20,21,22,23,24,25]. Thus, for CHC patients treated with antiviral therapies, maintaining skeletal muscle mass is crucial. However, to the best of our knowledge, there have been no available data with regard to changes in skeletal muscle mass for CHC patients with SVR who underwent IFN-free DAAs therapy. Further, identifying pretreatment factors linked to the improvement in skeletal muscle mass may be pivotal for creating nutritional strategies in CHC patients after SVR.

The aims of the current study were therefore to examine changes in skeletal muscle mass in CHC patients undergoing IFN-free DAAs therapy who achieved SVR and to identify pretreatment predictors that are associated with the improvement in skeletal muscle mass.

## 2. Patients and Methods

### 2.1. Patients and Skeletal Muscle Mass Measurement

This study was a single center retrospective study. All study participants were CHC patients with data for skeletal muscle mass at baseline and SVR. Patients without those data were excluded from the current analysis.

For all analyzed subjects, skeletal muscle mass was assessed using bio-impedance analysis (BIA, Inbody720, Takumi Ltd., Aichi, Japan) in the standing position. BIA is a device which can measure the body fat mass, body water mass, and body muscle mass using differences in frequency [11].

Our current study participants were CHC patients treated with IFN-free DAAs therapy (DCV/ASV, SOF/LDV, SOF/LDV/RBV, SOF/RBV, or others) who were admitted at the Division of Hepatobiliary and Pancreatic disease, Department of Internal Medicine, Hyogo College of Medicine, Hyogo, Japan between August 2013 and August 2015. All patients achieved SVR and had available data for BIA. In this analysis, SVR was defined as the disappearance of serum HCV-RNA at a time point more than 12 weeks after the completion of each DAA therapy. In principal, BIA was routinely performed at baseline and at SVR 24 (24 weeks after the completion of DAAs therapy). All patients had no ascites on radiologic findings. Skeletal muscle index (SMI) was defined as the sum of skeletal muscle mass in the upper and lower extremities divided by height squared (cm^2^/m^2^), using data from BIA [11]. We compared the changes in SMI at baseline and SMI at SVR. Patients with increased SMI were defined as those with an SMI at SVR that was more than the SMI at baseline. Further, we identified pretreatment parameters contributing to the increased SMI using univariate and multivariate analyses. Included pretreatment parameters (potentially relevant factors with the development of SML in view of current published articles) are listed in Table 1 [9,11,12,13,14]. The ethical committee meeting at Hyogo College of Medicine approved our current study protocol and this study strictly followed all provisions of the Declaration of Helsinki.

### 2.2. Statistical Analysis

First, the distribution of each parameter (normal or not) was assessed by the Shapiro-Wilk test. Categorical variables were compared by Fisher’s exact test. Continuous variables were compared by unpaired *t*-test, paired *t*-test, or Mann-Whitney *U* test as applicable. For predicting increased SMI, candidate variables were identified by univariate analysis; variables with *p* < 0.10 were analyzed by a multivariate logistic regression analysis. Data are presented as median value (range) unless otherwise mentioned. Statistical significance was set at *p* < 0.05. Statistical analysis was performed with JMP 11 (SAS Institute Inc., Cary, NC, USA).

## 3. Results

### 3.1. Baseline Characteristics

Baseline characteristics in the present study are shown in Table 1. Our study cohort (*n* = 69; 55 in HCV genotype 1 and 14 in genotype 2) included 31 males and 38 females with a median (range) age of 63 (25–83) years. SMI in males at baseline ranged from 6.73 to 9.08 cm^2^/m^2^ (median, 7.65 cm^2^/m^2^), while SMI in females at baseline ranged from 4.45 to 7.27 cm^2^/m^2^ (median, 5.81 cm^2^/m^2^). According to current guidelines, the proportion of low SMI in males (<7.0 cm^2^/m^2^) was 22.6% (7/31) and that in females (<5.7 cm^2^/m^2^) was 44.7% (17/38) [11]. For the entire cohort, SMI had normal distribution.

### 3.2. Changes in SMI for the Entire Cohort (n = 69)

**Figure 1:**
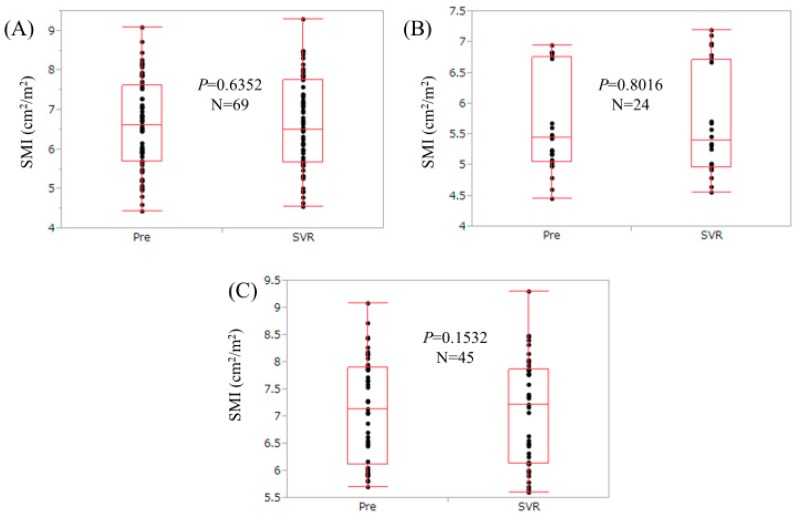
Changes in skeletal muscle index (SMI) during interferon (IFN)-free direct acting antivirals (DAAs) therapy at pretreatment and sustained virological response (SVR). (**A**) For the entire cohort (*n* = 69); (**B**) For patients with low muscle mass at baseline (*n* = 24, as defined by current guidelines [11]); (**C**) For patients without low muscle mass at baseline (*n* = 45, as defined by current guidelines [11]).

The median SMI for the entire cohort at baseline was 6.62 cm^2^/m^2^ (range, 4.45–9.08 cm^2^/m^2^), while the median SMI for the entire cohort at SVR was 6.51 cm^2^/m^2^ (range, 4.55–9.30 cm^2^/m^2^). (*p* = 0.6352, Figure 1A) The proportion of increased SMI at SVR compared with the SMI at baseline was 52.2% (36/69, 19 males and 17 females).

### 3.3. Changes in SMI for Patients with Low Muscle Mass (Low SMI) at Baseline (n = 24)

The median SMI for patients with low muscle mass (low SMI) at baseline was 5.44 cm^2^/m^2^ (range, 4.45–6.95 cm^2^/m^2^), while the median SMI for those patients at SVR was 5.40 cm^2^/m^2^ (range, 4.55–7.20 cm^2^/m^2^) (*p* = 0.8016, Figure 1B).

### 3.4. Changes in SMI for Patients without Low Muscle Mass (Low SMI) at Baseline (n = 45)

The median SMI for patients without low muscle mass (low SMI) at baseline was 7.13 cm^2^/m^2^ (range, 5.70–9.08 cm^2^/m^2^), while the median SMI for those patients at SVR was 7.22 cm^2^/m^2^ (range, 5.59–9.30 cm^2^/m^2^) (*p* = 0.1532, Figure 1C).

### 3.5. Changes in SMI According to Baseline FIB-4 Index

**Figure 2:**
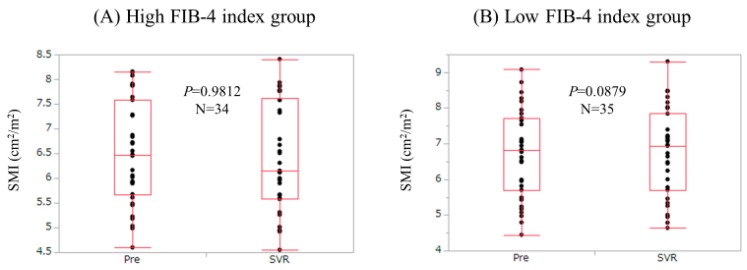
Changes in SMI according to baseline FIB-4 index. (**A**) Patients with baseline FIB-4 index ≥2.46 (the median value in our cohort) were defined as the high FIB-4 index group (*n* = 34). SMI at SVR did not significantly increase as compared with baseline levels (*p* = 0.9812); (**B**) Patients with baseline FIB-4 index <2.46 were defined as the low FIB-4 index group (*n* = 35). SMI at SVR tended to significantly increase as compared with baseline levels (*p* = 0.0879).

We compared changes in SMI according to FIB-4 index. Patients with baseline FIB-4 index ≥2.46 (the median value in our cohort) were defined as the high FIB-4 index group (*n* = 34), while those with baseline FIB-4 index <2.46 were defined as the low FIB-4 index group (*n* = 35). In the high FIB-4 index group, SMI at SVR did not significantly increase as compared with baseline levels (*p* = 0.9812). In the low FIB-4 index group, SMI at SVR tended to significantly increase as compared with baseline levels (*p* = 0.0879) (Figure 2).

### 3.6. Changes in SMI According to HCV Serotype

**Figure 3:**
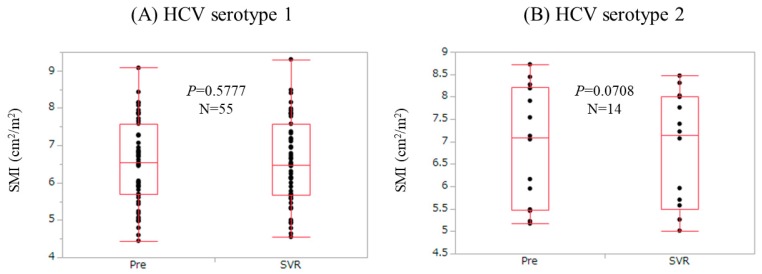
Changes in SMI according to HCV serotype. (**A**) In the HCV serotype 1 group (*n* = 55), SMI at SVR did not significantly increase as compared with baseline levels (*p* = 0.5777); (**B**) In the HCV serotype 2 group (*n* = 14), SMI at SVR tended to significantly decrease as compared with baseline levels (*p* = 0.0708).

In the HCV serotype 1 group (*n* = 55), SMI at SVR did not significantly increase as compared with baseline levels (*p* = 0.5777). In the HCV serotype 2 group (*n* = 14), SMI at SVR tended to significantly decrease as compared with baseline levels (*p* = 0.0708) (Figure 3).

### 3.7. Changes in SMI According to HCV Viral Load

**Figure 4:**
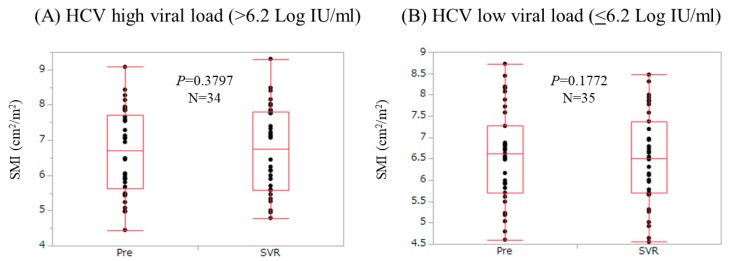
Changes in SMI according to HCV viral load. Patients with baseline HCV viral load >6.2 log IU/mL (the median value in our cohort) were defined as the high HCV viral load group (*n* = 34), while those with baseline HCV viral load ≤6.2 log IU/mL were defined as the low HCV viral load (*n* = 35). (**A**) In the high HCV viral load group, SMI at SVR did not significantly increase as compared with baseline levels (*p* = 0.3797); (**B**) In the low HCV viral load group, SMI at SVR did not significantly increase as compared with baseline levels (*p* = 0.1772).

We compared changes in SMI according to baseline HCV viral load. Patients with baseline HCV viral load >6.2 log IU/mL (the median value in our cohort) were defined as the high HCV viral load group (*n* = 34), while those with baseline HCV viral load ≤6.2 log IU/mL were defined as the low HCV viral load (*n* = 35). In the high HCV viral load group, SMI at SVR did not significantly increase as compared with baseline levels (*p* = 0.3797). In the low HCV viral load group, SMI at SVR did not significantly increase as compared with baseline levels (*p* = 0.1772) (Figure 4).

### 3.8. Changes in SMI According to Age

**Figure 5:**
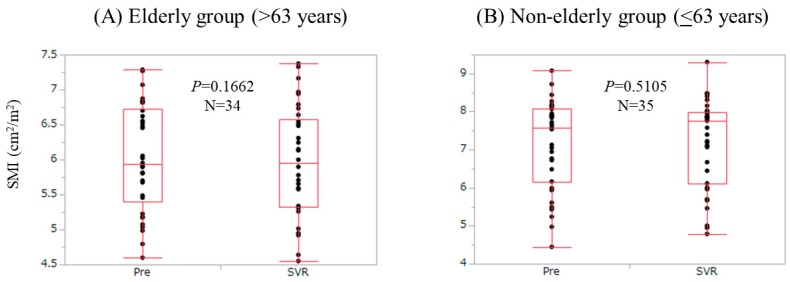
Changes in SMI according age. Patients with the age of >63 years (the median value in our cohort) were defined as the elderly group (*n* = 34), while those with the age of ≤63 years were defined as the non-elderly group (*n* = 35). (**A**) In the elderly group, SMI at SVR did not significantly increase as compared with baseline levels (*p* = 0.1662); (**B**) In the non-elderly group, SMI at SVR did not significantly increase as compared with baseline levels (*p* = 0.5105).

We compared changes in SMI according to age. Patients with the age of >63 years (the median value in our cohort) were defined as the elderly group (*n* = 34), while those with the age of ≤63 years were defined as the non-elderly group (*n* = 35). In the elderly group, SMI at SVR did not significantly increase as compared with baseline levels (*p* = 0.1662). In the non-elderly group, SMI at SVR did not significantly increase as compared with baseline levels (*p* = 0.5105) (Figure 5).

### 3.9. Comparison of Baseline Characteristics Between Patients with and without Increased SMI

Comparison of baseline characteristics between patients with increased SMI (*n* = 36) and without increased SMI (*n* = 33) is shown in Table 2. Age (*p* = 0.0392) and hyaluronic acid level (*p* = 0.0143) in the increased SMI group were significantly lower than those in the non-increased SMI group. Branched-chain amino acid to tyrosine ratio (BTR) in the increased SMI group was significantly higher than those in the non-increased SMI group (*p* = 0.0024). FIB-4 index in the increased SMI group tended to be significantly lower than that in the non-increased SMI group (*p* = 0.0656).

### 3.10. Multivariate Analyses of Factors Linked to the Presence of Increased SMI

Multivariate analysis for the above four factors with *p* < 0.10 (i.e., age, hyaluronic acid, FIB-4 index, and BTR) showed that only BTR was a significant prognostic pretreatment factor linked to the presence of increased SMI (*p* = 0.0488). (Table 3) Odds ratios and 95% confidence intervals are demonstrated in Table 3.

## 4. Discussion

Liver disease patients are aging in our country and SML is also associated with aging [11]. In other words, SML in liver disease patients can occur, irrespective of the degree of liver fibrosis stage. CHC therapy has dramatically improved due to the introduction of DAAs [1,4]. As described earlier, SML can be an adverse predictor in patients with liver diseases, although there are limited clinical data showing that skeletal muscle mass improvement can lead to favorable clinical outcomes in CHC patients, and reversing skeletal muscle mass loss is a priority field for therapeutic strategies in these patients [9,10,11,12,13]. In view of these backgrounds, addressing clinical queries to which factors are associated with the improvement of skeletal muscle mass after SVR may be a point of focus, and our current results may therefore be worthy of reporting.

In the multivariate analysis, only pretreatment BTR value was an independent predictor linked to increased SMI. BTR has also been shown to decrease in cirrhotic patients. Furthermore, BTR is widely used in Japan as an easily measurable indicator of amino acid imbalance and it is also closely related to protein synthesis in the muscle [9,26]. In addition, in our previous study, we demonstrated that lower BTR was associated with decreased skeletal muscle mass in patients with chronic liver diseases, which is in agreement with our current data [27]. In a sense, measuring BTR can lead to creating strategies for nutritional intervention from the view point of skeletal muscle mass. In patients with well-preserved protein synthesis ability, as reflected by a higher BTR level, HCV eradication may lead to the acceleration of protein synthesis in the muscle; this is a major finding in the current study. On the contrary, considering our current results, patients with LC status are expected to have poor improvement in skeletal muscle mass even after HCV eradication. Patients with a higher baseline FIB-4 index had poor improvement in skeletal muscle mass at SVR in our results, and this observation can support our hypothesis. In such patients, some interventions including exercise may be recommended [9,12,13]. In compensated LC patients, walking 5000 or more steps per day may be ideal [28]. On the other hand, it is of note that the proportion of low SMI in males was rather lower than that in females (22.6% vs. 44.7%). Significant differences in baseline characteristics between males and females (age; *p* < 0.0001, FIB-4 index; *p* = 0.0196, BTR; *p* = 0.0292, data not shown) may account for our current results.

It is of interest that in the HCV serotype 2 group, SMI at SVR tended to significantly decrease as compared with baseline levels (*p* = 0.0708). One possible reason for these results is that in HCV serotype 2 patients, RBV was used in most cases. RBV-related anemia may cause muscle mass decrease.

Whether SMI increment presents a good prognosis in chronic liver disease patients has yet to be clarified. On the other hand, previous investigators reported that a higher BTR was a favorable predictor for LC patients [29]. In our results, higher BTR was associated with SMI increment. In view of this, SMI increment in CHC patients can result in a good prognosis. However, further studies will be required to confirm these results.

Our study had the drawbacks of a small sample size for analysis, and how these short-term outcomes translate into long-term outcomes has yet to be determined. In addition, several important variables that could influence the outcome were overlooked (diet, exercise, and other pharmacological therapies), as they were not included in the current analysis. The causal relationship between BTR and skeletal muscle mass should also be elucidated. Thus, further well-designed and larger studies with longer observation periods will be needed in the future. However, our results denote that pretreatment BTR level is a useful predictor for improvement in skeletal muscle mass after SVR in CHC patients treated with DAAs.

## 5. Conclusions

In conclusion, pretreatment BTR levels can be helpful for predicting increased SMI after SVR in CHC patients undergoing IFN-free DAAs therapy.

## Figures and Tables

**Table 1:** Baseline data (*n* = 69).

Variables	Number or Median (Range)
Age (years)	63 (25–83)
Gender, male/female	31/38
Body mass index (kg/m^2^)	22.1 (15.7–32.8)
Skeletal muscle index (cm^2^/m^2^), male	7.65 (6.73–9.08)
Skeletal muscle index (cm^2^/m^2^), female	5.81 (4.45–7.27)
Total bilirubin (mg/dL)	0.8 (0.3–3.0)
Serum albumin (g/dL)	4.1 (2.8–4.9)
Prothrombin time (%)	83.4 (61.1–119.4)
Platelets (×10^4^/mm^3^)	15.5 (3.3–27.8)
Serum sodium (mmol/L)	140 (129–144)
eGFR (mL/min/1.73 m^2^)	84 (33–142)
Total cholesterol (mg/dL)	159 (110–234)
Triglyceride (mg/dL)	88 (33–779)
AST (IU/L)	37 (15–140)
ALT (IU/L)	37 (11–155)
Fasting blood glucose (mg/dL)	94 (74–187)
HbA1c (NSGP)	5.5 (4.1–9.7)
BTR	4.94 (2.13–9.09)
Alpha-fetoprotein (ng/mL)	4.2 (1.3–224.9)
Hyaluronic acid (ng/mL)	103 (9–699)
FIB-4 index	2.46 (0.66–20.04)
HCV genotype, 1/2	55/14
HCV viral load (log IU/L)	6.2 (5.0–7.7)

Data are expressed as number or median (range). eGFR; estimated glomerular filtration rate, AST; aspartate aminotransferase, ALT; alanine aminotransferase, NGSP; National Glycohemoglobin Standardization Program, BTR; branched-chain amino acid to tyrosine ratio, HCV; hepatitis C virus.

**Table 2:** Comparison of baseline characteristics between patients with increased SMI (I-SMI, *n* = 36) and without I-SMI (*n* = 33).

Variables	I-SMI (*n* = 36)	Non-I-SMI (*n* = 33)	*p* Value
Age (years)	59 (25–78)	65 (39–83)	0.0392
Gender, male/female	18/18	13/20	0.4693
Serum albumin (g/dL)	4.1 (3.3–4.9)	4.1 (2.8–4.6)	0.6883
Total bilirubin (mg/dL)	0.8 (0.3–2.5)	0.7 (0.4–3.0)	0.3416
Prothrombin time (%)	84.05 (67–108.4)	82.8 (61.1–119.4)	0.4161
Platelet count (×10^4^/mm^3^)	17.35 (3.6–25.3)	14.2 (3.3–27.8)	0.3433
AST (IU/L)	29.5 (15–140)	45 (19–120)	0.2735
ALT (IU/L)	36.5 (11–155)	39 (13–104)	0.4971
Serum sodium (mmol/L)	140 (129–144)	141 (135–143)	0.7724
Total cholesterol (mg/dL)	158 (110–228)	159 (126–234)	0.5347
Triglyceride (mg/dL)	92 (33–174)	88 (45–779)	0.9091
Fasting blood glucose (mg/dL)	93 (74–187)	96 (85–130)	0.1224
eGFR (mL/min/1.73 m^2^)	85 (33–141)	82 (36–142)	0.8803
HbA1c (NSGP)	5.5 (4.7–9.7)	5.5 (4.1–7.0)	0.4911
Body mass index (kg/m^2^)	21.95 (15.7–30.8)	22.5 (17.4–32.8)	0.3988
BTR	5.16 (3.01–9.09)	4.19 (2.13–8.36)	0.0024
Hyaluronic acid (ng/mL)	80 (9–437)	156 (17–699)	0.0143
FIB-4 index	1.94 (0.66–20.04)	3.53 (0.96–15.29)	0.0656
Alpha-fetoprotein (ng/mL)	3.1 (1.9–224.9)	7.3 (1.3–183.3)	0.4694
HCV genotype, 1/2	31/5	24/9	0.2331
HCV-RNA (log IU/L)	6.3 (5.1–7.2)	6.2 (5.0–7.7)	0.9380

AST; aspartate aminotransferase, ALT; alanine aminotransferase, eGFR; estimated glomerular filtration rate, NGSP; National Glycohemoglobin Standardization Program, BTR; branched chain amino acid to tyrosine ratio, HCV; hepatitis C virus. Patients with I-SMI indicate those with an SMI at SVR the was more than the SMI at baseline.

**Table 3:** Multivariate analysis of factors contributing to the increased SMI.

Variables	Multivariate Analysis	
	OR (95% CI)	*p* Value
Age (per one year)	1.029 (0.977–1.089)	0.2797
Hyaluronic acid (per one ng/mL)	1.002 (0.998–1.006)	0.3794
BTR (per one)	0.648 (0.398–0.998)	0.0488
FIB-4 index (per one)	0.996 (0.832–1.221)	0.9688

OR; Odds ratio, CI; confidence interval, BTR; branched-chain amino acid to tyrosine ratio.

## Abbreviations

CHC	chronic hepatitis C
HCV	hepatitis C virus
SVR	sustained virological response
DAA	direct acting antiviral
SMV	Simeprevir
Peg-IFN	pegylated interferon
RBV	Ribavirin
DCV	Daclatasvir
ASV	Asunaprevir
SOF	Sofosbuvir
LDV	Ledipasvir
SML	skeletal muscle loss
LC	liver cirrhosis
BIA	bio-impedance analysis
SMI	skeletal muscle index
BTR	branched-chain amino acid to tyrosine ratio
